# Two old drugs, NVP-AEW541 and GSK-J4, repurposed against the *Toxoplasma gondii* RH strain

**DOI:** 10.1186/s13071-020-04094-2

**Published:** 2020-05-11

**Authors:** Shuxian Liu, Mimi Wu, Qianqian Hua, Daiqiang Lu, Yuan Tian, Helin Yu, Linyan Cheng, Yinqi Chen, Jiaxin Cao, Xin Hu, Feng Tan

**Affiliations:** 1 Department of Parasitology, School of Basic Medical Sciences, Wenzhou Medical University, Wenzhou, 325035 Zhejiang China; 2 Clinical Laboratory, Dongyang People’s Hospital, Jinhua, 322100 Zhejiang People’s Republic of China; 3 School of the First Clinical Medical Sciences, Wenzhou Medical University, Wenzhou, 325035 Zhejiang China; 4 School of the Second Clinical Medical Sciences, Wenzhou Medical University, Wenzhou, 325035 Zhejiang China; 5 School of Ophthalmology & Optometry, Wenzhou Medical University, Wenzhou, 325035 Zhejiang China; 6 School of Medical Laboratory Science and School of Life Science, Wenzhou Medical University, Wenzhou, 325035 Zhejiang China

**Keywords:** NVP-AEW541, GSK-J4, *Toxoplasma gondii*, Invasion, Intracellular replication, *In vivo*

## Abstract

**Background:**

*Toxoplasma gondii* is a zoonotic pathogen that causes toxoplasmosis and leads to serious public health problems in developing countries. However, current clinical therapeutic drugs have some disadvantages, such as serious side effects, a long course of treatment and the emergence of drug-resistant strains. The urgent need to identify novel anti-*Toxoplasma* drugs has initiated the effective strategy of repurposing well-characterized drugs. As a principled screening for the identification of effective compounds against *Toxoplasma gondii*, in the current study, a collection of 666 compounds were screened for their ability to significantly inhibit *Toxoplasma* growth.

**Methods:**

The inhibition of parasite growth was determined using a luminescence-based β-galactosidase activity assay. Meanwhile, the effect of compounds on the viability of host cells was measured using CCK8. To assess the inhibition of the selected compounds on discrete steps of the *T. gondii* lytic cycle, the invasion, intracellular proliferation and egress abilities were evaluated. Finally, a murine infection model of toxoplasmosis was used to monitor the protective efficacy of drugs against acute infection of a highly virulent RH strain.

**Results:**

A total of 68 compounds demonstrated more than 70% parasite growth inhibition. After excluding compounds that impaired host cell viability, we further characterized two compounds, NVP-AEW541 and GSK-J4 HCl, which had IC_50_ values for parasite growth of 1.17 μM and 2.37 μM, respectively. In addition, both compounds showed low toxicity to the host cell. Furthermore, we demonstrated that NVP-AEW541 inhibits tachyzoite invasion, while GSK-J4 HCl inhibits intracellular tachyzoite proliferation by halting cell cycle progression from G1 to S phase. These findings prompted us to analyse the efficacy of the two compounds *in vivo* by using established mouse models of acute toxoplasmosis. In addition to prolonging the survival time of mice acutely infected with *T. gondii*, both compounds had a remarkable ability to reduce the parasite burden of tissues.

**Conclusions:**

Our findings suggest that both NVP-AEW541 and GSK-J4 could be potentially repurposed as candidate drugs against *T. gondii* infection.
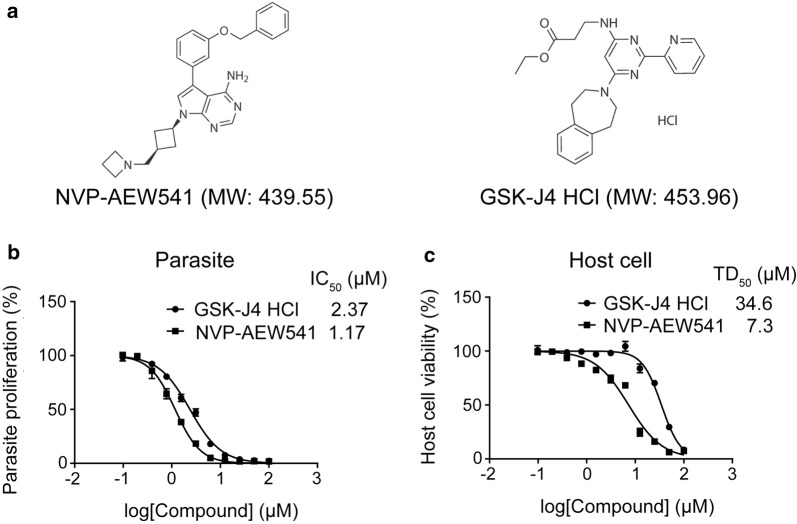

## Background

Toxoplasmosis, caused by the obligate intracellular parasite *Toxoplasma gondii*, is a ubiquitous zoonotic parasitic disease worldwide. This parasite has low host specificity and infects almost all warm-blooded mammals and birds, including nearly one-third of the human population [1]. Humans and other hosts become infected by ingestion of either tissue cysts in undercooked meat or oocysts shed in felid faeces. While asymptomatic in healthy individuals, the disease may cause significant morbidity and mortality primarily in individuals who are either immunocompromised, such as HIV-infected patients and immunosuppressed organ transplant patients, or congenitally infected foetuses [2, 3]. In addition, the associations between *Toxoplasma* infection and neurological disorders, such as schizophrenia, have been made in several investigations [4–7]. Moreover, *T. gondii* infection may induce abortion and stillbirth in livestock, which causes significant reproductive losses and, as a result, economic losses [8]. Meanwhile, animals infected with *T. gondii* are also an important source of infection. Studies reported that toxoplasmosis causes the highest disease burden of foodborne pathogens in developed countries, and ultimately is the second leading cause of death due to foodborne illness [9]. Therefore, *T. gondii* infection is becoming a worldwide public health problem.

*Toxoplasma* infection is usually handled by the immune response of the host and administered drugs. However, only a limited number of drugs are available for the clinical treatment of toxoplasmosis, including spiramycin and the combination of pyrimethamine and sulfadiazine as well as the combination of trimethoprim and sulfamethoxazole. Second-line therapy drugs include atovaquinone, clindamycin and epiroprim [10, 11]. Additionally, some research groups have developed a diverse of array of promising new compounds against *T. gondii*, such as calcium-dependent protein kinase 1 inhibitors [12], endochin-like quinolones [13], bumped kinase inhibitors [14] and artemisinine derivatives [15]. However, despite the effective treatment of toxoplasmosis, these chemotherapies often have several disadvantages: (i) the therapeutic effect of these drugs is limited because approximately 50% of patients infected with *T. gondii* do not respond to the treatment [10]; (ii) patient tolerance can be poor because of serious side effects, including bone marrow suppression, allergic reactions, agranulocytosis and megaloblastic anaemia [16, 17]; (iii) resistance to these drugs has emerged, and vaccines are thus far from futile [18, 19]; and (iv) some off-patent clinical drugs, such as pyrimethamine, are becoming increasingly expensive, which makes it difficult for some poor nations and populations to obtain [20, 21]. Therefore, it is necessary to identify safe, effective and affordable anti-*T. gondii* drugs.

Screening an unbiased compound library is rapidly becoming a common approach to identify effective candidates to treat parasitic infection in the early stages of drug development [22–24]. However, drug development is an expensive and long-term process due to the lack of information about host toxicity and the actual mechanism of action of compounds. Repurposing well-characterized drugs and compounds that are already approved for the treatment of other conditions could overcome these challenges [25–28]. One of the advantages of this strategy is that the pharmacokinetic and pharmacodynamic parameters have been established for each compound, and their assumed targets are also known. Therefore, these characteristics are expected to accelerate the novel clinical applications of well characterized drugs [29, 30]. In this study, we screened a library of 666 FDA approved compounds to identify those that suppress *T. gondii* growth *in vitro* and *in vivo*.

## Methods

### Chemicals

The Selleck New Compound Library, consisting of 666 compounds (each stored as a 10 mM stock solution in DMSO), was obtained from the Shanghai Institute of Biochemistry and Cell Biology of Chinese Academy of Sciences, China (http://www.sibcb.ac.cn/cp13-5_3.asp), and used for preliminary screens against *Toxoplasma*. For further studies, both NVP-AEW541 and GSK-J4 HCl were obtained from Selleck (Cat# S7070 and S1034, respectively). Pyrimethamine (Cat# 46706, Sigma-Aldrich, Shanghai, China) was included in the experiments as a reference drug. A stock solution of these compounds was prepared in 100% dimethyl sulfoxide (DMSO, Sigma-Aldrich), stored at − 80 °C, and diluted in fresh culture medium prior to each use, with a final DMSO concentration below 0.1%. A23187 (Cat# 100105, Sigma-Aldrich) was dissolved in DMSO at 1 mM and stored at − 20 °C.

### Parasites and host cells

The *T. gondii* tachyzoites were maintained by repeat passage in monolayers of human foreskin fibroblasts (HFFs, ATCC SCRC-1041) cells grown in Dulbecco’s modified Eagleʼs medium (DMEM; Gibco, Invitrogen, Shanghai, China) supplemented with 10% (v/v) foetal bovine serum (FBS; Gibco, Invitrogen) and a cocktail of 1% (v/v) penicillin-streptomycin-glutamine (Cat# 10378016, Gibco) at 37 °C and 5% CO_2_.

### Small-molecule preliminary screen

In the preliminary screening, the inhibition of parasite growth was determined using a luminescence-based β-galactosidase (β-Gal) activity assay as previously described [24, 28, 31]. Briefly, small molecular compounds were freshly reconstituted in culture medium to final concentrations of 5 μM and added to confluent monolayers of HFFs plated in 96-well half-area plates. Then, freshly purified RH-2F tachyzoites [32], which express β-Gal, were added at a multiplicity of infection (MOI) of 0.2 (parasite/host cell ratio). Infected HFFs treated with 0.1% DMSO were served as a negative control, and 10 μM pyrimethamine, a concentration sufficient to show the maximum inhibitory effect for parasite growth [23], was used as a reference compound to serve as a positive control. After a 72 h incubation at 37 °C and 5% CO_2_, parasite replication was stopped by the addition of 1% Triton X-100, and β (β)-Gal activity was determined following the addition of 1 mM chlorophenol red-β-d-galactopyranoside (CPRG; Cat# 220588, Sigma-Aldrich) and monitoring the absorption at 570 nm. The number of parasites was deduced according to the standard curves generated in parallel in each plate.

To measure the effect of the compounds that positively inhibited parasite growth (≥ 70% parasite inhibition) on the viability of host cells, 10 µl of Cell Counting Kit-8 Reagent (CCK8, Dojindo, Shanghai, China) was added to uninfected HFFs after 72 h of growth. The absorbance signal was recorded at 450 nm after the addition of CCK8 reagent for 2 h. After the compounds that inhibited parasite growth at less than 70% and/or showed obvious host cytotoxicity (≤ 80% cell viability) were eliminated, the drugs of interest were selected from the remaining compounds to further assess the drug efficacy.

### *In vitro* assessment of drug efficacy

To assess the efficacy of the selected compounds, NVP-AEW541 and GSK-J4 HCl, against *T. gondii*, the β-Gal activity was determined again as described previously [13]. Each selected compound with an initial concentration of 100 μM in the culture medium was added to the first column of HFFs (~ 250 cells/well) in a 96-well half-area plate and then diluted serially across the plate by 2-fold dilutions, leaving the final column drug-free. Therefore, the exact concentration was 100, 50, 25, 12.5, 6.25, 3.125, 1.56, 0.78, 0.39, 0.195, 0.098 and 0 μM, respectively. Fifty tachyzoites were then added at a MOI of 1:5 to each well in six of the eight rows. After a 72 h incubation, CPRG was added, and the absorbance was measured at 570 nm. Meanwhile, to measure the effect of each compound on the viability of host cells, CCK8 reagent was added to the two rows of uninfected HFFs and absorbance at 450 nm was measured after 2 h. The assay was performed in triplicate and repeated three times. Either the 50% toxicity dose (TD_50_) for the host cell, or the 50% inhibitory concentration (IC_50_) for the parasite was calculated, and the therapeutic index (TI) of each compound was defined as TD_50_/IC_50_. A high TI indicates that the compound specifically inhibits *T. gondii* replication and has a negligible effect on HFFs.

### Plaque assay

HFF monolayers in 24-well plates containing either NVP-AEW541 (1.25 μM) or GSK-J4 HCl (2.5 μM) in triplicate were infected with approximately 100 tachyzoites. After incubation for 7 days, the morphology and integrity of the monolayers were observed directly under a light microscope. Then, the infected monolayers were washed once with cold PBS, fixed with methanol for 10 min, stained with 1.5% crystal violet for 10 min, and washed once in PBS. The area of each plaque in a given well was measured and summed using ImageJ as a proxy for growth.

### Invasion assay

The invasion assay was performed as described previously [33] with modifications. RH GFP-TgAtg8 parasites [34] inoculated onto HFFs were pre-treated with NVP-AEW541 (2.5 μM), GSK-J4 HCl (5 μM) or vehicle (0.1% DMSO). At 8 h post-infection (hpi), the tachyzoites were lysed from host cells by passage through a 27-gauge needle and purified from host cell debris through a 3 μm filter (Whatman, GE Healthcare, Beijing, China). Freshly harvested and purified tachyzoites were added to HFFs at a MOI = 5 on coverslips in a 24-well plate, kept on ice for 30 min and incubated at 37 °C for 1 h. Following invasion, the medium was gently removed, and the cells were washed three times with ice cold PBS and fixed with 4% paraformaldehyde. Parasites were stained with mouse anti-*T. gondii* Surface Antigen 1 (SAG1, 1:200, Cat# C86319M; Meridian, TN, USA) and detected with anti-mouse Alexa Fluor 594 (1:1000, Invitrogen, Shanghai, China) without permeabilization of the cells. Nuclear DNA was labelled with DAPI for 5 min, and the coverslips were mounted with 50% glycerol. Intracellular parasites were scored as GFP^+^SAG1^−^, and extracellular parasites were scored as GFP^+^SAG1^+^. A total of 20 randomly selected fields were counted in triplicate from three independent biological replicates.

### Intracellular proliferation assay

To measure the ability of the two compounds to inhibit intracellular tachyzoite proliferation, HFFs on coverslips were infected with fresh extracellular GFP-TgAtg8 tachyzoites at a MOI = 4. After 2 h, the medium was replaced with medium containing NVP-AEW541 and GSK-J4 HCl at final concentrations of 2.5 μM and 5 μM, respectively. After incubation for 24 h, the slides were fixed and observed as described above. The number of parasites in each of at least 50 randomly chosen parasitophorous vacuoles was counted. Average numbers of parasites per vacuole were calculated.

### Egress assay

Monolayer HFFs on coverslips in 24-well plates were infected with purified GFP-TgAtg8 tachyzoites at a MOI = 5. At 6 hpi, the cells were washed to remove extracellular parasites and incubated in fresh medium containing the specified compound for 24 h. Calcium was induced with 3 μM A23187 for 5 min at 37 °C, and egress was stopped by the addition of cold 100% methanol for 10 min on ice. The proportion of egressed *versus* non-egressed vacuoles was calculated by counting at least 50 vacuoles.

### Flow cytometric analyses of the cell cycle

Flow cytometric analyses were performed as described previously [14, 35]. Briefly, HFFs were infected with GFP-TgAtg8 tachyzoites at a MOI = 1. At 12 hpi, the cell cycle was synchronized by adding 80 μM pyrrolidine dithiocarbamate (PDTC, Sigma-Aldrich) and incubating at 37 °C and 5% CO_2_ for 8 h [36]. After removing the PDTC, cells were incubated at 37 °C and 5% CO_2_ for 8 h in media containing the specified compound. The extracellular parasites were removed by washing with ice-cold PBS, and the intracellular tachyzoites were isolated by passage of host cells through a 27-gauge needle and purified with a 3 μm filter. The purified parasites were fixed with ice cold ethanol at − 20 °C for 24 h, and their DNA was stained by using propidium iodide (PI) solution (40 μg/ml PI, 360 μg/ml RNase A and 2% BSA in PBS) at 37 °C for 30 min in the dark. The stained parasites were filtered with a 5 μm pore filter and analysed on a FACSCanto™II flow cytometer (BD Biosciences, CA, USA). The results were analysed on an EPICS-XL flow cytometer (Beckman Coulter, CA, USA) by using SYSTEM II software (Beckman Coulter), and the percentages of the population in each phase of the cell cycle (G1, S, and G2 +M) were calculated [37]. At least 10,000 events were collected per sample.

### Murine infection model of toxoplasmosis

All mice were purchased from the Laboratory Animal Center, Wenzhou Medical University, Zhejiang, China. They were bred and handled in strict accordance with the Good Animal Practice requirements of the Animal Ethics Procedures and Guidelines of China. In a preliminary experiment aiming to assess the side effects of compounds, female BALB/c mice (6–8 weeks old, ~20 g) were orally administered with each compound at 50 mg/kg for 5 days and were monitored for 30 days. There was no obvious clinical toxicity after treatment with all compounds during this time period.

To evaluate the ability of the two compounds to protect against acute *T. gondii* infection *in vivo*, we used a BALB/c murine model and challenged the mice with the RH-2F strain of *T. gondii*. Mice were divided into 4 groups consisting of 15 mice each and injected intraperitoneally with 10^3^ RH strains per animal. Compounds were dissolved in DMSO and diluted in PBS prior to feeding to mice. After 4 h of infection, mice were treated for 5 consecutive days with daily oral administration of the specified compound at 50 mg/kg (once a day). Mice treated with pyrimethamine (50 mg/kg) were used as a positive control, and 1 ml PBS containing 11 μl of DMSO was used as a negative control. Within each group, 5 mice were euthanized at 7 dpi for comparison of parasite burden, and 10 mice were part of a 20-day survival experiment. Mice were monitored as described previously [38] and scored twice per day for signs of toxoplasmosis, such as impaired mobility, difficulty in feeding, weight loss, self-mutilation and severe ascites. When any mice showed the above signs, reached the maximum tolerable score for 2 consecutive days and were assessed as having no possibility of recovery, humane endpoints were used to avoid animal pain or suffering *via* euthanasia as requested by the Animal Ethics Procedures and Guidelines of China. At the end of the experimental period, all remaining mice were euthanized.

Meanwhile, the parasite burden in the brain, liver and spleen of infected mice was measured at 7 dpi as described previously [27, 39]. Briefly, whole organs were mixed in lysis buffer (50 mM Tris pH 8.0, 50 mM EDTA, 1% SDS, 50 mg/ml of proteinase K) and incubated overnight at 56 °C. Total genomic DNA from 25 mg of tissue in 100 μl of lysis buffer was then extracted using DNeasy Blood and Tissue Kit (Qiagen, Shanghai, China) following the manufacturer’s instructions. The *Toxoplasma* B1 gene was detected by quantitative PCR (qPCR) with the primers TgB1-F (5′-GGA GGA CTG GCA ACC TGG TGT CG-3′) and TgB1-R (5′-TTG TTT CAC CCG GAC CGT TTA GCA G-3′) [40]. Amplification was performed in a final volume of 20 μl using a ChamQ Universal SYBR qPCR Master Mix (Cat# Q711-02; Vazyme, Nanjing, China). Each reaction contained 10 μl of 2× ChamQ SYBR qPCR Master Mix, 1 μl of template DNA, 8.2 μl of distilled water and 0.4 μM each primer. The following amplification conditions were applied: 30 s at 95 °C; 40 cycles of 95 °C for 10 s, 60 °C for 30 s; and one cycle of 95 °C for 15 s, 60 °C for 1 min and 95 °C for 15 s. The number of parasites was calculated from the standard curve prepared in parallel.

### Statistical analysis

Data were plotted using Prism 7.0 (GraphPad Software Inc., San Diego, CA, USA) and are presented as the mean of three replicates ± standard deviation (SD). The TD_50_ and IC_50_ values were plotted through a nonlinear regression analysis (curve fit). Statistical analysis was carried out with one-way ANOVA and Tukey’s multiple comparison test for all *in vitro* data as well as *in vivo* parasite burden. The survival rate was analysed by using the Kaplan-Meier method. The overall difference between survival curves was tested for statistical significance using the asymptotic log-rank test for multiple groups based on a chi-square probability distribution with 3 degrees of freedom (*df*). Dunnett’s multiple comparisons test was used to compare the survival time of mice among different groups. *P*-value < 0.05 was considered statistically significant.

## Results

### Small molecule screen to identify known compounds that inhibit *T. gondii* growth

**Fig. 1:**
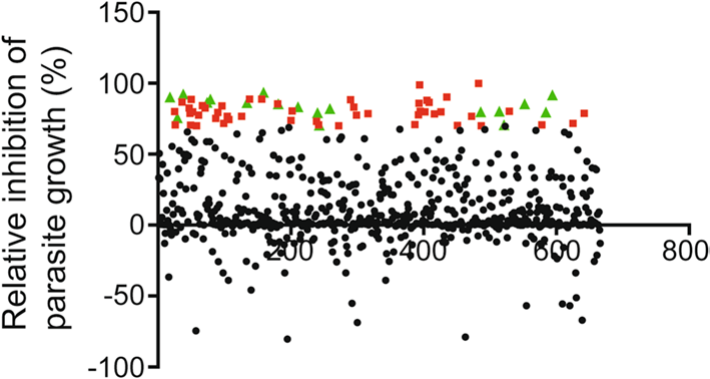
Primary small-molecule screening identifies compounds that inhibit *Toxoplasma* growth. RH-2F-infected HFF cells were incubated with 5 μM each compound for 72 h. β-Gal activity was detected; and inhibition was calculated with DMSO as a negative control (0% inhibition) and 10 μM pyrimethamine as a positive control (100% inhibition). Furthermore, compounds that showed more than 70% parasite inhibition (68 compounds) were screened for their effects on host cell viability. Green triangles, no host cell toxicity; red rectangles, host cell toxicity; black circles, less than 70% parasite inhibition

The Selleck New Compound Library, which is a collection of 666 FDA-approved small-molecule compounds, was screened to identify the *T. gondii* growth inhibitory effects. In a preliminary screening, β-Gal-expressing RH strain tachyzoites were used to infect HFFs pre-treated with each compound at 5 μM. At 72 hpi, β-Gal activity was detected by the addition of CPRG. Within each plate, a standard curve was made and used for calculation of the parasites in each well. A total of 68 compounds inhibited parasite growth by at least 70% (Additional file 1: Table S1). Furthermore, host cell viability assays showed that 18 compounds did not induce obvious host cell cytotoxicity (≥ 80% cell viability; Fig. 1, Table 1), suggesting that the inhibitory effect of these compounds on parasite growth was not induced by host cell cytotoxicity.

**Table 1:** Eighteen hit compounds from preliminary screening

Compound	Parasite inhibition (%) Mean ± SD	Host viability (%) Mean ± SD
Atazanavir	93.6 ± 2.1	90.7 ± 2.8
PHA-665752	92.3 ± 3.1	80.5 ± 1.3
NVP-AEW541	91.5 ± 2.5	80.7 ± 1.2
Cyclosporine	90.8 ± 2.1	90.4 ± 1.4
GSK-J4 HCl	90.4 ± 1.4	92.2 ± 3.8
Pemetrexed disodium	88.9 ± 3.6	84.6 ± 1.5
Mocetinostat	86.5 ± 3.1	80.2 ± 3.8
AMG-208	86.4 ± 5.3	82.8 ± 1.8
CHR2797	85.6 ± 1.8	88.8 ± 6.7
H 89 2HCl	83.5 ± 1.8	89.4 ± 4.6
Blonanserin	82.1 ± 3.1	88.3 ± 5.9
Lurasidone HCl	80.4 ± 1.5	86.9 ± 1.7
ZM 323881 HCl	79.9 ± 1.4	85.5 ± 5.4
Pamidronate disodium	79.6 ± 1.5	80.8 ± 1.4
Cyproheptadine HCl	79.2 ± 3.5	90.6 ± 1.2
Entinostat	75.7 ± 2.2	81.6 ± 1.5
Medetomidine HCl	70.8 ± 1.5	90.5 ± 4.4
Lafutidine	70.6 ± 2.5	92.7 ± 5.6

*Abbreviation*: SD, standard deviation

### Validation of the antiparasitic activity of NVP-AEW541 and GSK-J4

**Fig. 2:**
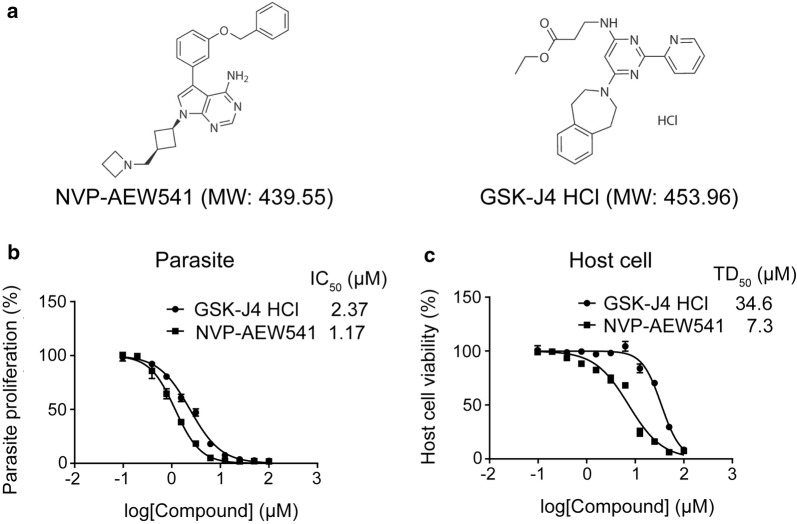
NVP-AEW541 and GSK-J4 HCl display dose-dependent inhibitory effects on *T. gondii* growth. **a** Structures and molecular weights of NVP-AEW541 and GSK-J4 HCl. **b** Dose-response response curves and IC_50_ values for NVP-AEW541 and GSK-J4 HCl. RH-2F parasite-infected HFFs were incubated with serial dilutions (0 to 100 μM) of the test compounds, and β-Gal activity was measured. **c** Dose-response response curves and TD_50_ values for NVP-AEW541 and GSK-J4 HCl. HFFs were incubated with serial dilutions (0 to 100 μM) of the test compounds, and cell growth was measured by using CCK8

Of the five compounds with the highest inhibitory efficiency (≥ 90% parasite inhibition), three have been reported to inhibit the growth of *T. gondii* [28, 41]. Thus, the remaining two compounds, NVP-AEW541 and GSK-J4 HCl, were further analysed at various concentrations to verify their antiparasitic activity and to determine their therapeutic index (TI). First, dose-dependent parasite growth inhibition curves were obtained for the two compounds (Fig. 2b). The results showed that NVP-AEW541 and GSK-J4 HCl inhibit *T. gondii* at IC_50_ values of 1.17 μM and 2.37 μM, respectively. Moreover, host cell viability was measured using CCK8 reagent to assess host cell cytotoxicity, which may further influence the antiparasitic activity. This measurement also provides an initial indication of potential human cytotoxicity. The TD_50_ value of NVP-AEW541 against HFF was 7.3 μM, while the TD_50_ value of GSK-J4 HCl against HFF was 34.6 μM. Based on these results, the calculated *in vitro* TI was 6.2 for NVP-AEW541 and 14.6 for GSK-J4 HCl. These data suggest that NVP-AEW541 and GSK-J4 HCl are potent drug candidates against toxoplasmosis.

### Plaque assay verifying that both NVP-AEW541 and GSK-J4 impair the lytic cycle of tachyzoites

**Fig. 3:**
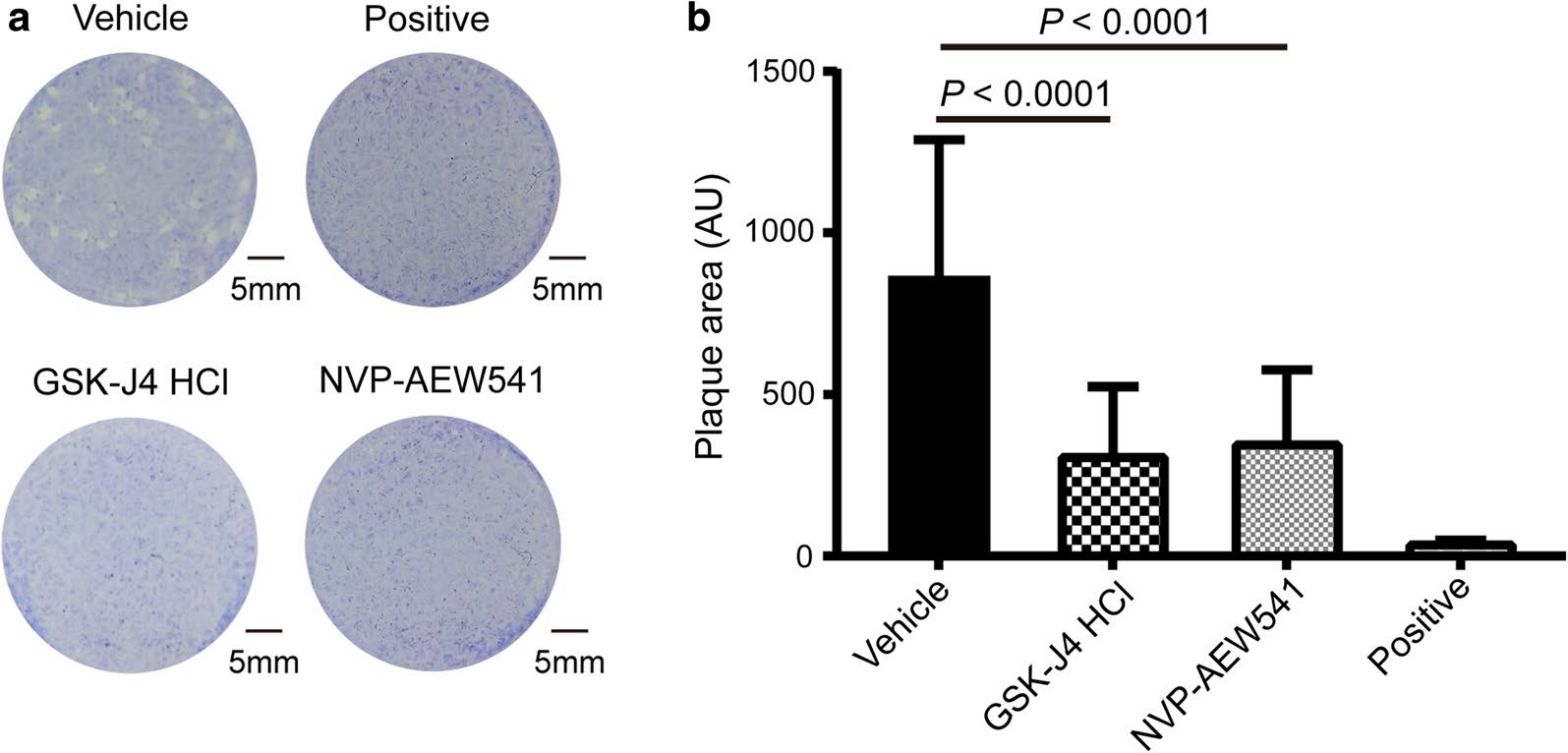
Effects of NVP-AEW541 and GSK-J4 HCl on the lytic cycle of parasites. **a** Plaque assays were carried out by infecting HFF monolayers with RH parasites treated with NVP-AEW541 (1.25 μM), GSK-J4 HCl (2.5 μM), DMSO or pyrimethamine for 7 days. **b** Lysis plaque areas were measured. Shown are the average values and standard deviations from one experiment representative of three independent experiments. *Abbreviation*: AU, arbitrary units

To verify whether the two compounds are active against *T. gondii*, plaque assays were used first. Based on the screening results, tachyzoites within infected monolayers were treated with each compound at the IC_50_ value for 7 days. As anticipated, an overall statistically significant difference between groups was demonstrated (*F*_(3, 157)_ = 40.74, *P* < 0.0001). The parasites treated with NVP-AEW541 and GSK-J4 HCl exhibited approximately 60% and 65% reductions in plaque area compared with the vehicle control, respectively (Fig. 3), suggesting that the two compounds indeed inhibit the replication of tachyzoites. In addition, visual inspection showed that the monolayer was intact after treatment with the two compounds.

### NVP-AEW541 inhibits *Toxoplasma* invasion

These results encouraged us to assess the importance of the two compounds in discrete steps of the *T. gondii* tachyzoite lytic cycle, including host cell invasion, intracellular proliferation, and egress. First, we found that neither NVP-AEW541 nor GSK-J4 HCl inhibits *Toxoplasma* egress (*F*_(2, 6)_ = 1.396, *P* = 0.3179).

**Fig. 4:**
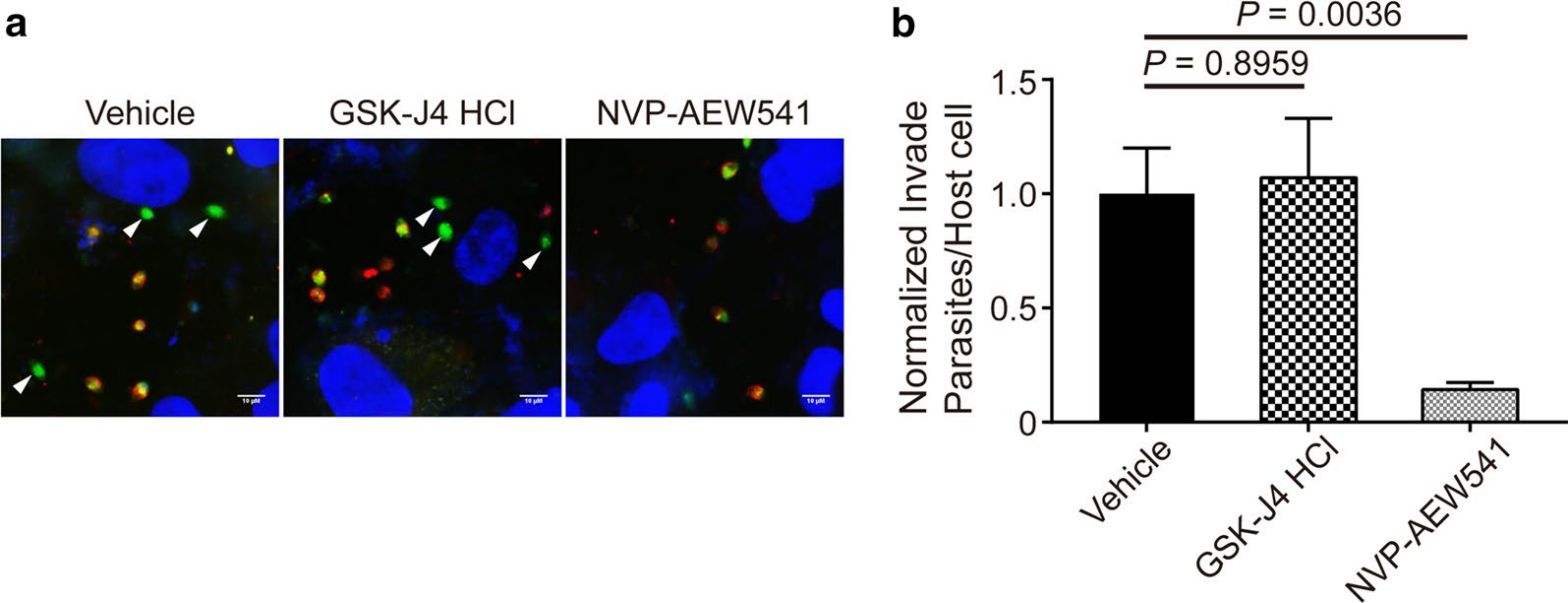
Inhibitory effects of NVP-AEW541 and GSK-J4 HCl on parasite invasion. RH GFP-TgAtg8-infected HFFs were pre-treated with NVP-AEW541 (2.5 μM), GSK-J4 HCl (5 μM) or DMSO for 8 h, and then the tachyzoites were released and added to fresh HFFs to invade for 1 h. The cells were fixed without permeabilization and stained with DAPI and anti-SAG1 antibody. **a** Representative immunofluorescence micrographs of parasites treated with vehicle (DMSO), GSK-J4 HCl and NVP-AEW541, respectively. White arrowheads indicate the intracellular parasites. **b** Invasion events were scored *via* differential staining as GFP^+^SAG1^−^ (invaded) and GFP^+^SAG1^+^ (extracellular). A total of 20 randomly selected fields were counted for each triplicate sample. Shown are the average values and standard deviations from one experiment representative of three independent experiments

To test whether the two compounds affect parasite invasion, intracellular RH GFP-TgAtg8 cells that could express green fluorescence and were no different from the RH wildtype strain in the lytic cycle [34] were utilized. Additionally, before evaluation of the lytic cycle using this strain, we also observed the inhibitory effect of two compounds on it. The IC_50_ value was completely consistent with that obtained by using RH-2F strain. In the invasion assay, RH GFP-TgAtg8 was pre-treated with each compound or the vehicle control for 8 h, and freshly egressed tachyzoites were added to the host cell for assessment of invasion ability. After 1 h, the cells were fixed but not permeabilized and then stained with anti-SAG1 antibody to discriminate between extracellular (GFP^+^SAG1^+^) and intracellular (GFP^+^SAG1^−^) parasites. Statistical analysis showed that there was an overall significant difference in the number of intracellular parasites between groups (*F*_(2, 6)_ = 22.09, *P* = 0.0017). We found that NVP-AEW541 significantly decreased the number of intracellular parasites by approximately 85% (Fig. 4), indicating that the drug affected parasite invasion. In contrast, the ability of parasites to invade host cells was not affected by GSK-J4 HCl.

### GSK-J4 inhibits the cell cycle and intracellular growth of *Toxoplasma*

**Fig. 5:**
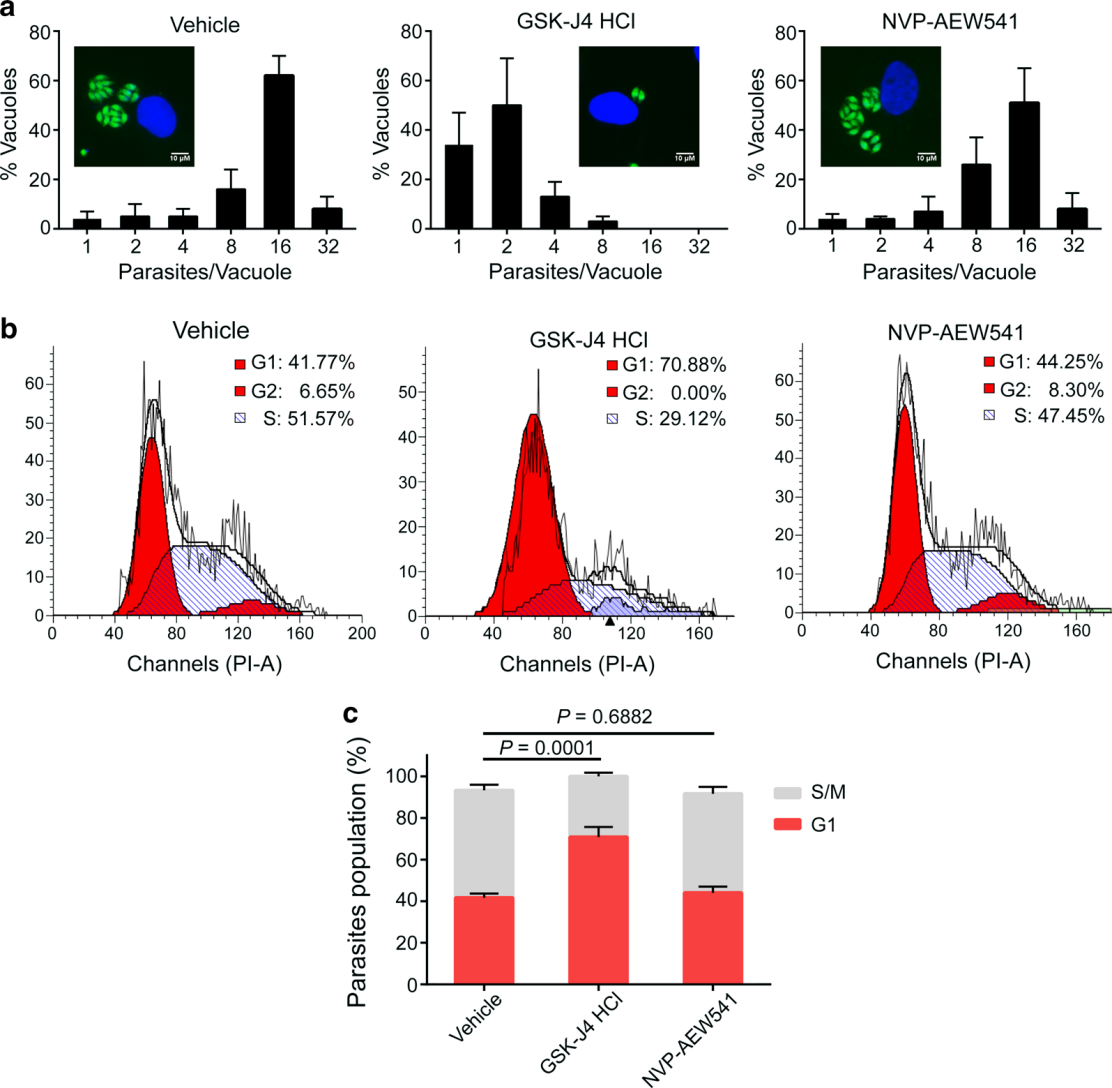
Inhibitory effects of NVP-AEW541 and GSK-J4 HCl on intracellular parasite replication. **a** HFFs were treated with NVP-AEW541 (2.5 μM), GSK-J4 HCl (5 μM) or DMSO for 24 h following infection with RH GFP-TgAtg8 parasites for 2 h. The cells were fixed, and the number of parasites per vacuole was determined. In addition, representative immunofluorescence micrographs of intracellular parasites are shown in the corresponding bar chart. **b** Flow cytometric analysis of the effects of NVP-AEW541 and GSK-J4 HCl on cell cycle progression. The DNA contents of the parasites were measured by PI staining. **c** The percentage of parasites in the S/M and G1 phases of the cell cycle

To test whether the two compounds are active against intracellular growth, tachyzoites were allowed to invade host cells for 2 h. The extracellular parasites were then removed by washing the cells, and fresh medium containing one of the two compounds or vehicle was added. The cells were fixed 24 h later, and the number of tachyzoites per vacuole was counted by observing GFP-positive parasites. In contrast to cells with DMSO-treated parasites that largely consisted of vacuoles containing 8 and 16 tachyzoites, GSK-J4 HCl significantly inhibited *Toxoplasma* intracellular growth, as shown by the accumulation of vacuoles with mostly one or two tachyzoites (Fig. 5a). In contrast, *Toxoplasma* intracellular growth was not impaired by NVP-AEW541.

To clarify how GSK-J4 HCl affects intracellular parasite growth, we investigated the effect of GSK-J4 HCl on parasite cell cycle progression. Flow cytometry was performed to analyse the cell cycle populations of G1 and S/M by quantitating the DNA contents in the single parasite. After the cell cycles of intracellular parasites were synchronized by PDTC treatment, intracellular parasites were released and grown for 8 h in the presence of DMSO, NVP-AEW541 or GSK-J4 HCl. An overall statistically significant difference in the population of G1 between groups was demonstrated (*F*_(2, 6)_ = 63.7, *P* < 0.0001). Furthermore, we noticed that parasites treated with GSK-J4 HCl, not NVP-AEW541, showed G1 accumulated peaks compared to the vehicle-treated control (Fig. 5b), indicating that GSK-J4 HCl delayed or arrested the cell cycle of the parasite by halting progression from G1 to S phase.

### Efficacy of NVP-AEW541 and GSK-J4 against acute murine toxoplasmosis

**Fig. 6:**
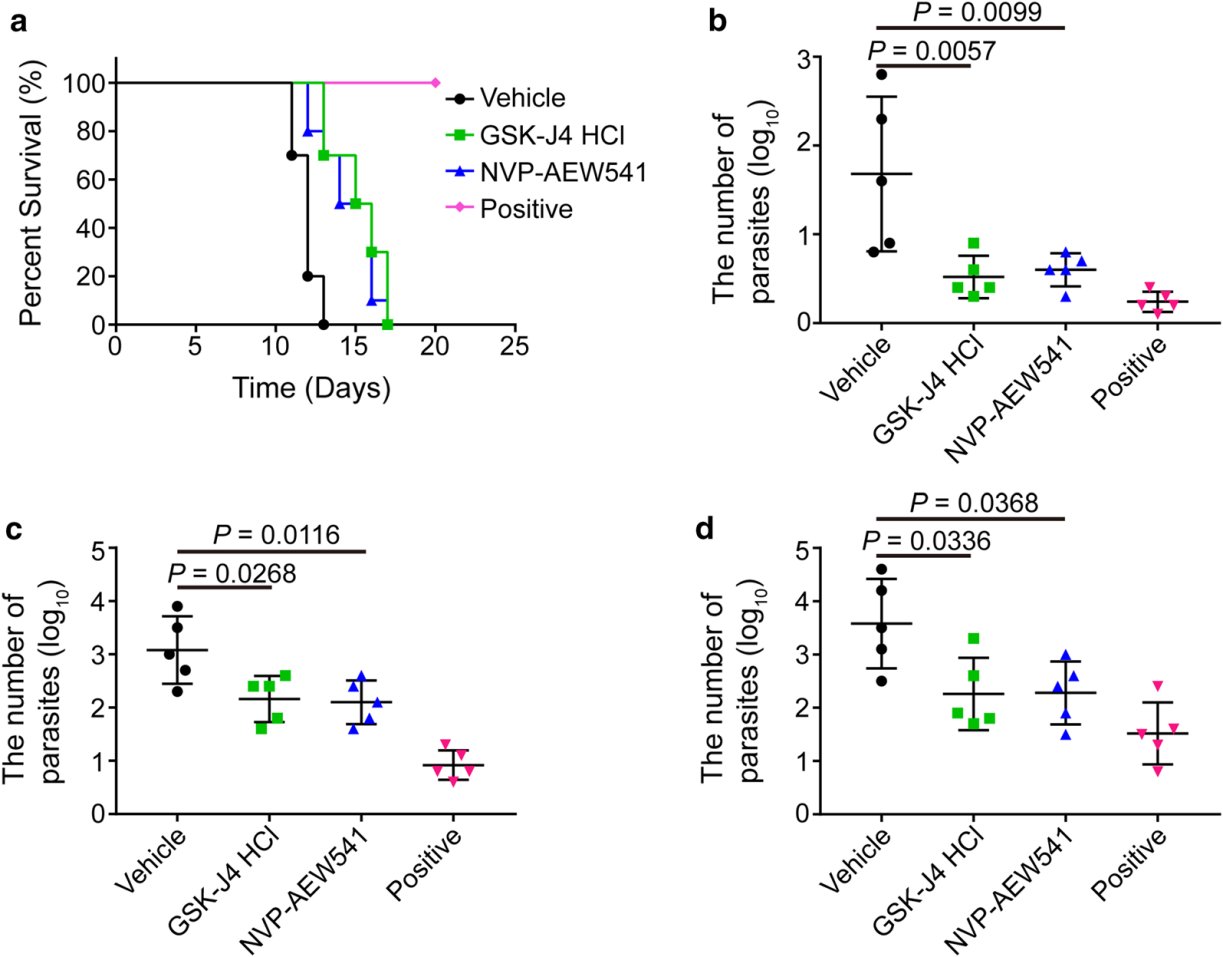
Protective efficacy of NVP-AEW541 and GSK-J4 HCl on acutely infected mice. **a** Effect of NVP-AEW541 and GSK-J4 HCl on the survival rate of infected mice. BALB/c mice were infected intraperitoneally (ip) with 100 RH strain tachyzoites. Beginning on the day of infection, each drug at 50 mg/kg, vehicle (DMSO) or positive drug (pyrimethamine) was orally administered for 5 days. These mice were observed for an additional 15 days, and the survival times of the infected mice were recorded for 20 days (*n* = 10 for each group). **b**–**d** Effect of NVP-AEW541 and GSK-J4 HCl on parasite burden in tissues of infected mice. At 7 dpi, qPCR analysis of infected brain (**b**), liver (**c**) and spleen (**d**) samples was performed by using the *Toxoplasma* B1 gene as an indicator of parasite burden. Error bars represent the averages and standard deviations for parasite burden among the mice examined (*n* = 5 per group)

Due to the good anti-*Toxoplasma* activity of the two compounds *in vitro*, we sought to determine whether they function against toxoplasmosis *in vivo*. We used a well-established model to monitor the protective efficacy of drugs against acute infection of highly virulent RH strain tachyzoites in laboratory mice. First, we found that all the healthy mice treated with each compound and pyrimethamine survived (data not shown), indicating that the two compounds do not lead to the death of uninfected mice under the present experimental conditions. Then, BALB/c mice were infected with RH tachyzoites and administered with either NVP-AEW541 or GSK-J4 HCl at 50 mg/kg of body weight. Drug treatment was initiated on the day of infection and administered for the next 4 days. Kaplan-Meier survival curves were plotted to present the survival rate of mice for each group (Fig. 6a). A log-rank test demonstrated an overall statistically significant difference in survival between all groups of mice (*χ*^2^ = 50.8, *df* = 3, *P* < 0.0001). Pairwise comparisons revealed that in comparison to the vehicle control group (11.9 ± 0.74 days), mice treated with NVP-AEW541 (14.6 ± 1.84 days, *P* = 0.008) and GSK-J4 HCl (15.2 ± 1.69 days, *P* = 0.001) had a significantly longer survival time.

The DNA copy number of parasites from brain, liver and spleen tissue lysates was detected at 7 dpi by using B1-based real-time PCR for quantitative assessment of parasite burden. The pattern of differences in mean parasite burden between groups changed significantly in the brain (*F*_(3, 16)_ = 9.281, *P* = 0.0009), liver (*F*_(3, 16)_ = 18.73, *P* < 0.0001) and spleen (*F*_(3, 16)_ = 7.912, *P* = 0.0018). As shown in Fig. 6b–d, although pyrimethamine-treated mice showed the lowest parasite burden in tissue, mice treated with each compound also had a significantly lower parasite burden compared with the vehicle control group.

## Discussion

In recent years, repurposing drugs or screening validated compound libraries to identify novel chemical entities for the treatment of toxoplasmosis has attracted increasing attention. Additionally, considering the obligate intracellular parasitism of *T. gondii* and its preference for cystic formation in brain tissue, small-molecule compounds have received increasing attention in the identification of anti-*T. gondii* drugs because of their lower molecular weight, better membrane permeability and higher specificity [22, 42–44]. Therefore, in the present investigation, we screened one FDA library comprising 666 small-molecule compounds to identify those that inhibited *T. gondii* growth. Although we used a known anti-*Toxoplasma* agent, pyrimethamine, to serve as a positive control, we also noticed that the library contained some established anti-*Toxoplasma* compounds, such as atazanavir [41], cyclosporine and PHA665752 [28], that, as expected, significantly inhibited *T. gondii* growth. On the other hand, we found that several compounds, such as SB-408124, semaxanib and U0126, had no effect on *T. gondii* growth, which is consistent with previous findings [28]. Therefore, these data demonstrated the relative robustness of our screen.

A total of 18 compounds from the library were identified as having strong antiparasitic activities and no apparent cytotoxicity. Among these compounds that we identified, we focused on two compounds, NVP-AEW541 and GSK-J4 HCl, which have not been previously reported to have anti-*Toxoplasma* activity. Importantly, they showed a higher inhibitory effect on *T. gondii* (≥ 90% parasite inhibition) and lower toxicity to host cells (≥ 80% cell viability). We investigated the potential of NVP-AEW541 and GSK-J4 HCl against *T. gondii in vitro* and *in vivo*. To our knowledge, we demonstrated for the first time that both NVP-AEW541 and GSK-J4 HCl have potent anti-*Toxoplasma* activity with low cytotoxic effects on human host cells *in vitro*, exhibiting promising TI values of 6.2 and 14.6, respectively. In another previous investigation, sulfadiazine and pyrimethamine, which are currently used in the clinical treatment of toxoplasmosis, have been shown to have lower TI values of ≤ 1 and ≤ 8, respectively [22]. Taken together, these findings suggest that the two compounds, especially GSK-J4 HCl, may be desirable agents for *T. gondii* therapy.

NVP-AEW541 is an orally bioavailable small-molecule inhibitor of insulin-like growth factor-1 receptor (IGF-1R) [45]. It belongs to the pyrrolo[2,3-d]pyrimidine class and has been identified to have a highly selective inhibitory effect on IGF-1R tyrosine kinase activity [46]. In our study, we showed that NVP-AEW541 effectively inhibits parasite growth by blocking tachyzoites from invading host cells. The invasion of *T. gondii* is a highly coordinated process in which the parasite begins to invade through an interaction between unknown parasite and host cell membrane surface factors. Then, the secretion of calcium-dependent micronemal proteins is induced, which acts as an adhesin to promote the formation of tight attachment between parasites and the host cell. Eventually, the parasite passes through the surface of the host cell and enters the cell to form nascent parasitophorous vacuoles [46]. Mechanistic studies indicated that NVP-AEW541 could induce cancer cell death by blocking cell cycle progression through caspase-dependent and caspase-independent pathways [45, 47–49], which have not been proven to mediate the invasion of *T. gondii* into host cells. Thus, we could not determine whether the drug hinders *Toxoplasma* invasion by inhibiting either a host or a parasite target. However, considering that IGF-1R, the target of NVP-AEW541, is a cell surface receptor, we speculate that IGF-1R may be involved in the process of *T. gondii* invasion, and further study will address these issues.

GSK-J4, a small-molecule inhibitor with highly efficient cell permeability [50], is a pharmacologically selective inhibitor that preserves histone H3 lysine 27 (H3K27) methylation by inhibiting histone lysine demethylase 6B (KDM6B), also known as Jumonji domain-containing protein D3 (JMJD3). The inhibitor acts by interacting with α-ketoglutarate binding at the catalytic site of KDM6B [50, 51]. It has been reported that the inhibition of histone demethylase by GSK-J4 suppresses embryonic development [52] and cellular differentiation [53]. In addition, treatment with GSK-J4 induced cell cycle arrest and cell death in different kinds of cancer cells with dismal toxicity to normal cells [54–58]. Likewise, our phenotype experiments also revealed that GSK-J4 effectively inhibits the intracellular proliferation of *T. gondii* by inducing tachyzoite cell cycle arrest. Interestingly, no clear orthologue of human KDM6B was found in the genome of *T. gondii* through homology searches in the ToxoDB database (https://toxodb.org/toxo/). In contrast, the homologue of KDM5B, another target of GSK-J4, which catalyses demethylation of histone 3 lysine 4 (H3K4) [59, 60], appears to be present in *T. gondii* (TGGT1_307010), suggesting that GSK-J4 HCl might have a somewhat similar antiparasitic mechanism.

Based on these findings *in vitro*, we sought to determine the efficacy of the two compounds against acute toxoplasmosis in a mouse model. Several previous studies demonstrated that at a dose of 50 mg/kg of body weight, no apparent toxicity was observed in experimental mice treated with NVP-AEW541 for 1–2 weeks [45, 61] or GSK-J4 HCl for 4–6 weeks [62]. Therefore, the same dose was used to treat mice in this study and did not lead to the death of healthy mice. However, we observed that compared with the vehicle control group, although the administration of the two compounds could significantly prolong the survival time of mice acutely infected with *T. gondii*, all the mice had to be euthanized due to signs of clinical toxoplasmosis during the 20-day experimental observation period. In contrast, all the mice in the positive drug treatment group survived. This limited effect observed *in vivo* suggests that drug treatment conditions, such as dose, time and route of administration, need to be further optimized. Nevertheless, the parasite burden in the brain, liver and spleen diminished significantly after treatment with the two compounds, indicating that NVP-AEW541 and GSK-J4 HCl are orally available drugs that are effective against acute murine toxoplasmosis.

While the two compounds are well characterized, it remains unclear whether they affect the parasite by a known mechanism of action. Therefore, further identification of the action targets of the two compounds against *T. gondii* is clearly warranted. To date, definite pharmacokinetic parameters of the two compounds have not been reported yet. However, one research revealed that NVP-AEW541 could inhibit NWT-21 cells (NIH3T3 stably expressing the human IGF-IR) with IC_50_ value of 1.64 μM *in vitro* and had anti-NWT-21 fibrosarcoma activity at the dose of 50 mg/ml in mouse model [45]. Another study showed that GSK-J4 HCl had a significant inhibitory effect on Kasumi-1 cells with IC_50_ value of 5.52 μM *in vitro* and decreased leukemia infiltration at the dose of 50 mg/ml in an AML xenograft mouse model by transplanting Kasumi-1 cells [62]. Notably, these studies not only demonstrate that they had no evident adverse effects *in vivo*, but also indicated that the plasma levels of the two compounds in mice could reach at least their respective IC_50_ values *in vitro*. Nevertheless, subsequent investigation of NVP-AEW541 and GSK-J4 HCl still needs to include broader pharmacokinetics and toxicological studies, as well as optimization of drug delivery routes and schedules for the eradication of *T. gondii* infection. Additionally, it would be valuable to further increase the drug concentration or use a longer treatment time to evaluate whether these compounds can act parasiticidal or only parasitostatic. Finally, future studies will also evaluate the synergistic effect of the two compounds with other clinically used anti-*Toxoplasma* drugs.

## Conclusions

Our study identified 18 compounds from a library of FDA approved compounds that potently inhibit *T. gondii* growth and have no host cytotoxicity. These findings are novel and promising, as well as further enhance the importance and feasibility of drug repurposing and screening of a wide range of small-molecule compounds as effective anti-toxoplasmosis treatments. Although further investigation into the mechanism of action against *T. gondii* is clearly needed, the present experiments suggest that NVP-AEW541 and GSK-J4 HCl are promising candidates for the treatment and prevention of toxoplasmosis.

## Supplementary information

**Additional file 1: Table S1.** Summarized information of inhibition of *Toxoplasma gondii* growth and host cell viability by all 666 compounds.

## Data Availability

Data supporting the conclusions of this article are included within the article and its additional file. The datasets used and/or analyzed in this study are available from the corresponding author upon request.

## Abbreviations

DMSO: dimethyl sulfoxide
DMEM: Dulbecco’s Modified Eagle Medium
HFFs: human foreskin fibroblasts
FBS: fetal bovine serum
β-Gal: β-galactosidase
MOI: multiplicity of infection
CPRG: chlorophenol red-β-d-galactopyranoside
CCK8: cell counting kit-8
TD_50_: 50% toxicity dose
IC_50_: 50% inhibitory concentration
TI: therapeutic index
hpi: hour post-infection
SAG1: surface antigen 1
PDTC: pyrrolidine dithiocarbamate
PI: propidium iodide
qPCR: quantitative PCR
IGF-1R: insulin-like growth factor-1 receptor
H3K27: histone 3 lysine 27
H3K4: histone 3 lysine 4
KDM6B: lysine demethylase 6B
JMJD3: Jumonji domain-containing protein D3
SD: standard deviation
